# A giant gastrointestinal stromal tumour mimicking gastric diverticulum: a case report

**DOI:** 10.1093/jscr/rjag172

**Published:** 2026-03-21

**Authors:** Sujit Kanta Mainali, Japana Regmi, Utsab Man Shrestha, Romeo Kansakar, Migma Shakya

**Affiliations:** Department of Laparoscopic and General Surgery, Madhyapur Hospital, Bhaktapur 44800, Nepal; Department of Medicine and Surgery, Madhyapur Hospital, Bhaktapur 44800, Nepal; Department of Oncosurgery, Bhaktapur Cancer Hospital, Bhaktapur 44800, Nepal; Department of GI and Hepatobiliary Surgery, Madhyapur Hospital, Bhaktapur 44800, Nepal; Department of Medicine and Surgery, Madhyapur Hospital, Bhaktapur 44800, Nepal

**Keywords:** gastrointestinal stromal tumor, imatinib, KIT mutation, gastric diverticulum, case report

## Abstract

Gastrointestinal stromal tumours (GISTs) are the most common mesenchymal tumors of the gastrointestinal tract, arising from the interstitial cells of Cajal. Although typically located in the stomach, they may present atypically, mimicking benign lesions such as gastric diverticula. This is a case of a 40-year-old Nepali female with rheumatoid arthritis and diabetes mellitus presenting with postprandial vomiting, abdominal fullness, and significant weight loss, where radiologic evaluation suggested a large posterior gastric diverticulum. On surgical excision, a gastric diverticulum was isolated, and histopathological analysis confirmed the diagnosis of a gastric GIST (C-kit+, DOG-1+, and CD34+). Despite low mitotic activity, the tumor was classified as high risk due to its size (>10 cm), and the patient was commenced on adjuvant imatinib therapy. This case highlights a gastric GIST masquerading as a giant gastric diverticulum and emphasizes the importance of considering GIST as a differential diagnosis of large gastric masses.

## Introduction

Gastrointestinal stromal tumours (GISTs) are rare mesenchymal neoplasms that arise predominantly in the stomach (56%), followed by the small intestine (32%), colon and rectum (6%), and esophagus (<1%) [1, 2]. They originate from the interstitial cells of Cajal or their precursors, which function as pacemaker cells for gastrointestinal motility. The annual incidence is estimated at 10–15 cases per million worldwide [3]. GISTs are immunohistochemically characterized by expression of KIT (CD117), also known as C-kit, and DOG-1, with additional positivity for CD34 (70%), SMA (30%–40%), and rarely desmin (<5%) or S100 (~5%) [4].

Although most GISTs are small and asymptomatic, large tumors within a gastric diverticulum may cause symptoms depending on the width of the diverticular neck. Wide-neck diverticula are usually asymptomatic, whereas narrow-neck diverticula may cause food retention, resulting in symptoms ranging from dyspepsia, epigastric pain, dysphagia, early satiety, and postprandial fullness to major gastrointestinal bleeding [5]. Atypical presentations may mimic benign pathologies, complicating diagnosis. We report a case of a giant gastric GIST initially misdiagnosed as a gastric diverticulum and managed successfully with surgical resection and adjuvant targeted therapy.

## Case presentation

**Figure 1:**
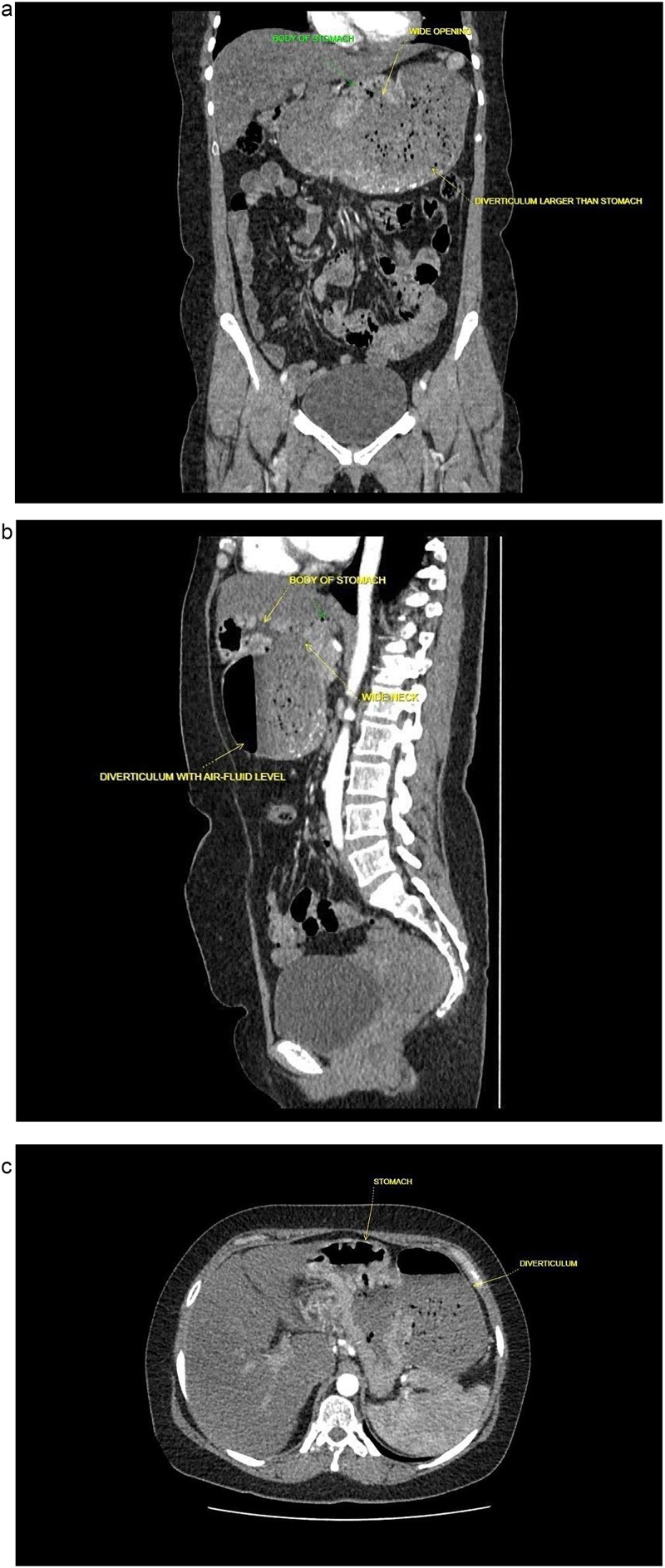
Contrast enhanced computed tomography (CECT) of abdomen and pelvis. (a) Sagittal plane. (b) Coronal plane. (c) Axial plane.

A 40-year-old postmenopausal female, known to have rheumatoid arthritis and type 2 diabetes mellitus, presented with persistent postprandial vomiting for 2 months, associated with abdominal fullness and weight loss (17 kg over 5 years). On physical examination, the patient was ill-looking but afebrile and haemodynamically stable. The abdomen was soft and non-tender, with mild epigastric fullness and no palpable mass. The timeline of clinical events from presentation to follow-up is shown in Table 1. Initial upper gastrointestinal endoscopy revealed retained food particles suggestive of gastric outlet obstruction. Ultrasonography was inconclusive. Contrast-enhanced CT of the abdomen and pelvis showed a large posteriorly located cystic lesion (18 × 11 × 12 cm) with internal debris, initially interpreted as a giant gastric diverticulum compressing adjacent structures (Fig. 1a–c).

**Table 1:** Timeline of clinical events.

Timeline	Clinical events
5 years prior	Unexplained weight loss of 17 kg over 5 years
2 months prior	Onset of postprandial vomiting and abdominal fullness
Initial visit	Endoscopy attempted-retained gastric contents
Hospital visits and admission	NG decompression: 1000–1200 ml, CECT suggested large gastric diverticulum from posterior wall of stomach
Surgery	Open excision of gastric diverticulum
Postoperative period	Uneventful recovery
Histopathology report in 1 week following operation	Suggested GIST, started on adjuvant therapy
On 6 month-follow-up	On adjuvant imatinib 400 mg daily; asymptomatic and disease free; plan to continue imatinib for 2.5 years

**Figure 2:**
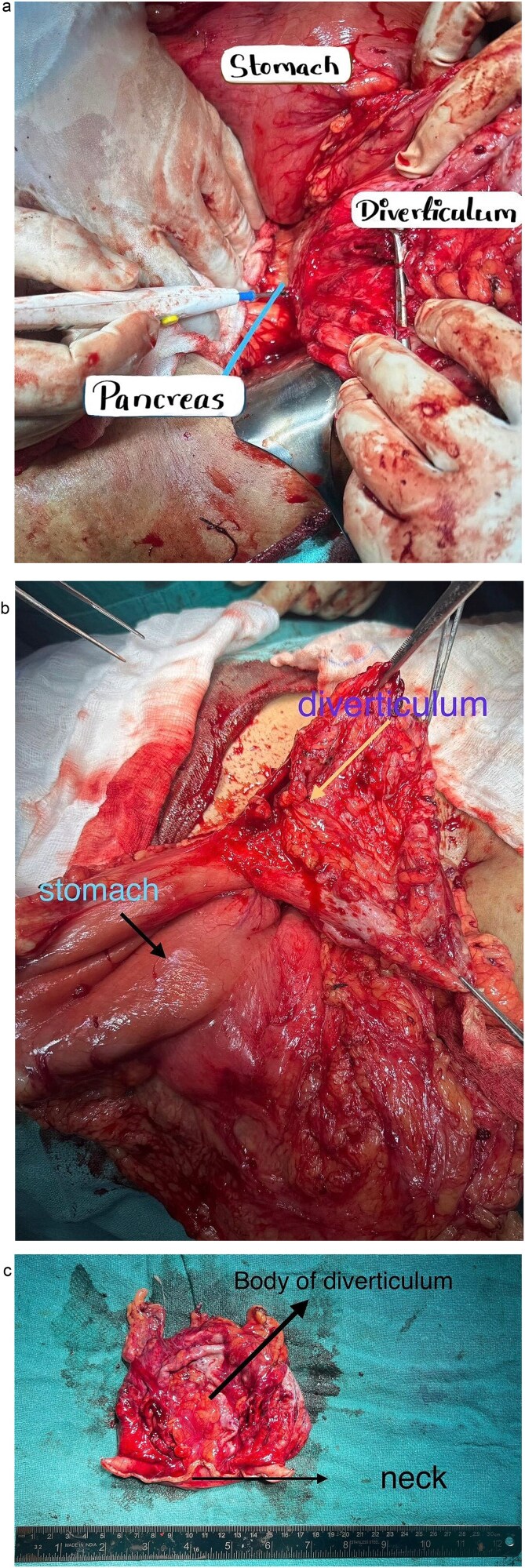
Intraoperative findings. (a) Gastric diverticulum attached to the posterior wall of stomach. (b) Gastric diverticulum attached to the posterior wall of stomach. (c) Gastric diverticulum following excision.

The patient underwent open surgical excision of the mass with 2 cm clear margins (Fig. 2a–c). No tumour rupture or peritoneal seeding was observed intraoperatively. The lesion appeared as a well-circumscribed, encapsulated mass arising from the posterior gastric wall with a large neck. Postoperatively, she received supportive care and was discharged on the seventh postoperative day. On one-week follow-up, immunohistochemistry confirmed spindle-shaped tumor cells arranged in fascicles with minimal pleomorphism and low mitotic activity (Table 2), consistent with a gastric GIST. Despite the low mitotic index, the tumor size (>10 cm) classified it as high risk according to the National Comprehensive Cancer Network criteria. Following multidisciplinary discussion, adjuvant imatinib (400 mg daily for 3 years) was initiated. At 6-month follow-up, the patient remains asymptomatic with no radiological evidence of recurrence or metastasis and continues imatinib with good tolerance.

**Table 2:** Immunohistochemistry profile.

C-kit: weak positive DOG-1: Positive CD34: Positive CK-8/18, Desmin: Negative Ki-67: 2%–3%
Consistent with GIST, spindle cell type, low-risk.

## Discussion

GIST behavior is primarily driven by mutations in the KIT gene (85%), PDGFRA gene (10%), or rarely the BRAF gene, with ~95% of tumours showing KIT positivity, leading to uncontrolled cellular proliferation in the gastrointestinal tract [6]. Clinically, GISTs often present with nonspecific gastrointestinal symptoms. Radiologically, their appearance varies depending on tumor size, growth pattern, and the presence of necrosis or hemorrhage, and they may mimic diverticular outpouchings or cystic lesions, increasing the likelihood of misinterpretation on imaging studies [7, 8]. In the present case, the cystic morphology of a posterior gastric wall GIST led to misdiagnosis as a gastric diverticulum.

Complete surgical resection with negative microscopic margins (R0 resection) remains the treatment of choice for localized GISTs and offers the best chance for long-term disease control. Open surgical approaches are preferred for tumors measuring ≥5 cm or those at risk of rupture to minimize intraoperative spillage and peritoneal dissemination [9]. For high-risk GISTs, evidence supports 3 years of adjuvant imatinib therapy, which significantly improves recurrence-free survival and overall prognosis compared with shorter regimens [10].

Immunohistochemical profiling further guide therapeutic decisions, as certain mutations may influence sensitivity to imatinib and require alternative dosing strategies or therapies [11, 12]. This case highlights the importance of accurate risk stratification and a multidisciplinary approach when large GISTs mimic benign gastric conditions on imaging.

## References

[1] Miettinen M, Lasota J. Gastrointestinal stromal tumors--definition, clinical, histological, immunohistochemical, and molecular genetic features and differential diagnosis. Virchows Arch 2001;438:1–12. 10.1007/s004280000338 11213830

[2] Søreide K, Sandvik OM, Søreide JA et al. Global epidemiology of gastrointestinal stromal tumours (GIST): a systematic review of population-based cohort studies. Cancer Epidemiol 2016;40:39–46. 10.1016/j.canep.2015.10.031 26618334

[3] Corless CL, Heinrich MC. Molecular pathobiology of gastrointestinal stromal sarcomas. Annu Rev Pathol 2008;3:557–86. 18039140 10.1146/annurev.pathmechdis.3.121806.151538

[4] Blay JY, Kang YK, Nishida T et al. Gastrointestinal stromal tumours. Nat Rev Dis Primers 2021;7:22. 10.1038/s41572-021-00254-5 33737510

[5] Shah J, Patel K, Sunkara T et al. Gastric diverticulum: a comprehensive review. Inflamm Intest Dis 2019;3:161–6. 10.1159/000495463 31111031 PMC6501548

[6] Demetri GD . Gastrointestinal stromal tumor. In: DeVita L, Lawrence TS, Rosenberg SA (eds). DeVita, Hellman, and Rosenberg’s Cancer: Principles and Practice of Oncology, 9th edn. Philadelphia: Wolters Kluwer Health/Lippincott Williams & Wilkins, 2011.

[7] Nishida T, Blay JY, Hirota S et al. The standard diagnosis, treatment, and follow-up of gastrointestinal stromal tumors based on guidelines. Gastric Cancer 2016;19:3–14. 10.1007/s10120-015-0526-8 26276366 PMC4688306

[8] Inoue A, Ota S, Yamasaki M et al. Gastrointestinal stromal tumors: a comprehensive radiological review. Jpn J Radiol 2022;40:1105–20. 10.1007/s11604-022-01305-x 35809209 PMC9616766

[9] Rutkowski P, Skoczylas J, Wisniewski P. Is the surgical margin in gastrointestinal stromal tumors different? Visc Med 2018;34:347–52. 10.1159/000491649 30498701 PMC6257087

[10] Joensuu H, Eriksson M, Sundby Hall K et al. Survival outcomes associated with 3 years vs 1 year of adjuvant Imatinib for patients with high-risk gastrointestinal stromal tumors: an analysis of a randomized clinical trial after 10-year follow-up. JAMA Oncol 2020;6:1241–6. 10.1001/jamaoncol.2020.2091 32469385 PMC7260691

[11] Casali PG, Blay JY, Abecassis N et al. ESMO-EURACAN-GENTURIS clinical practice guidelines for diagnosis, treatment and follow-up. Ann Oncol 2022;33:20–33. 10.1016/j.annonc.2021.09.005 34560242

[12] Rutkowski P, Gronchi A, Hohenberger P et al. Neoadjuvant imatinib in locally advanced gastrointestinal stromal tumors (GIST): the EORTC STBSG experience. Ann Surg Oncol 2013;20:2937–43. 10.1245/s10434-013-3013-7 23760587

